# Editorial: Model organisms and experimental models: opportunities and challenges in vascular physiology research

**DOI:** 10.3389/fphys.2023.1271220

**Published:** 2023-08-22

**Authors:** Victor M. Pulgar, Christelle Guibert, Al-Shaimaa Faissal Ahmed, Aaron J. Trask

**Affiliations:** ^1^ Department of Pharmaceutical and Clinical Sciences, Campbell University, Buies-Creek, NC, United States; ^2^ Department of Obstetrics and Gynecology, Wake Forest School of Medicine, Winston-Salem, NC, United States; ^3^ Université de Bordeaux, Institut National de la Santé et de la Recherche Médicale (INSERM) and Centre de Recherche Cardio-Thoracique de Bordeaux (CRCTB), U1045, Pessac, France; ^4^ Department of Pharmacology and Toxicology, Faculty of Pharmacy, Minia University, Minia, Egypt; ^5^ Center for Cardiovascular Research, The Research Institute at Nationwide Children’s Hospital, Columbus, OH, United States; ^6^ Department of Pediatrics, The Ohio State University College of Medicine, Columbus, OH, United States

**Keywords:** model organisms, experimental models, vascular physiology, vascular beds, blood flow regulation

## Introduction

Research is, at its core, a “search” that aims to understand “how.” In biomedical research, models allow access to simpler representations of the complex physiological systems we try to understand. Despite decades of “search” and significant understanding, cardiovascular diseases (CVD) continue to be highly prevalent, and better ways to approach complex physiology are needed. This Research Topic covers some of the complexities in vascular physiology, from the mechanisms involved in the generation of new vessels, the identification of biomarkers for CVD and the relationships between intrinsic and confounding factors in the determination of variables highly relevant for CVD population studies.

## Non-animal models to study angiogenesis

The generation of new blood vessels from pre-existing vessels is a complex process involving various cell types, coordinated cell-to-cell signaling pathways as well as mechanical influences, and it is an essential development for diverse areas such as CVD, oncology, and tissue regeneration. To study this process, several *in vivo*, *ex vivo*, and *in vitro* bioassays have been developed, each one with its inherent advantages and limitations (Nowak-Sliwinska et al., 2018). The review by Laschke et al. discussed several alternatives to *in vivo* experimentation that assess angiogenesis *in vitro,* such as endothelial cell viability assays and microfluidics assays that involve the study of cell migration, proliferation, and tube formation. Laschke et al. also presented a couple of *in vivo* models; the chorioallantois (CAM) assay that allows access to a highly vascularized membrane for the study of modulators of angiogenesis, and zebrafish embryos, which are not considered experimental animals before 120 h of development. This later model is gaining attraction in cardiovascular research in part because embryos are transparent, which facilitates observation and the following of angiogenesis. Along these lines, a recent review (Yuge et al., 2023) discussed the biochemical mechanisms of the dynamic process of angiogenesis, including the important roles of pericytes as regulators of endothelial cell proliferation. Among other processes, cardiac regeneration has also been studied in this model (Chowdhury et al., 2022). Zebrafish will certainly continue providing an efficient avenue for the study of cardiovascular development and disease mechanisms. Furthermore, as machine learning and artificial intelligence develop, the applications on modeling of CVD progression will also grow. The introduction of mathematical modeling of angiogenesis adds to our understanding of the applicability of these analyses combined with *in vivo* studies to better understand the process of sprouting and branching in angiogenesis (Tonami et al., 2023). The application of mathematical modeling extends to all aspects of CVD studies (Celant et al., 2023). Certainly, the development of novel models to study vascular diseases will continue to expand, providing avenues for high throughput analyses of diagnostic and therapeutic tools.

## Biomarkers in aneurysms

Perivascular adipose tissue (PVAT) comprises around 3% of all adipose tissue in the body. Initially thought to have mainly a mechanical support role, it is becoming clear that the endocrine nature of PVAT promotes multiple roles in the vasculature. These include controlling vascular tone and function through the release of a myriad of vasoactive substances (Nosalski and Guzik, 2017) to the modulation of the sympathetic-sensory interactions in perivascular nerves (Pulgar, 2020). PVAT functional and structural alterations have been linked to several vasculopathies, in addition to being a risk factor for the development of hypertension, atherosclerosis, diabetic vascular alterations, and aneurysms (Nosalski and Guzik, 2017). Abdominal aortic aneurisms (AAA) involve extensive aortic dilation with potentially fatal rupture, and it usually coexists with atherosclerosis. Alterations observed in AAA include inflammation, extracellular matrix degradation, vascular smooth muscle cell phenotypic modulation, and oxidative stress (Ye et al., 2021). Evidence linking PVAT with alterations in AAA shows increasing adipocytes in ruptured aortic walls in a mouse AAA model and a correlation between adventitial triglyceride levels and aorta diameter in human AAA (Kugo et al., 2016; Liu et al., 2023). In this Research Topic, Wang et al. aimed to identify biomarkers in PVAT that may predict AAA. Following a genetic analysis, several differentially expressed genes (DEGs) were identified in PVAT from AAA, mostly genes involved in neutrophil chemotaxis and IL-17 signaling pathways. The use of Gene Ontology (GO) and Kyoto Encyclopedia of Genes and Genomes (KEGG) analyses to explore the physiological pathways associated with the identified DEGs allowed the authors to identify five hub genes: *FOS*, *FOSB*, *EGR1*, *DUSP*, and *JUN*. Further analyses identified FOS as the most important characteristic gene in PVAT of AAA. These genetic findings were further explored *in vivo* using the AAA model of 4-weeks angiotensin II infusion in the ApoE^−/−^ mouse. Ang II treatment in this model produced increased aortic diameter and complete rupture of the aortic elastic fiber layer. Analyses in AAA tissue and non-dilated controls showed increased expression of FOS in AAA supporting its designation as an AAA biomarker. Since AAA at risk patients are mainly followed by imaging tools the search for biomarkers is of vital relevance, the article by Wang et al. adds to the attempts to have reliable AAA biomarkers, as has been recently described for some circulating biomarkers (Bouwens et al., 2023).

## Pulse Wave Velocity (PWV) and blood pressure: The case of reduced blood flow

Initial observations centuries ago suggested a relationship between the rate of cardiac beating (measured as pulse) and risk of diseases (Bedford, 1951). The transit of blood from the beating heart in systole to the tissues is greatly assisted by the elastic properties of the large vessels, such as the aorta, linking the heart and vessels, and conceptually making blood flow to tissues a continuous process. It has been known that this elastic role of large arteries deteriorates as we age and in several diseases in a process known as arterial stiffness (AS) (Townsend et al., 2015; Chirinos et al., 2019). PWV is the standard for measuring AS, and cumulative evidence shows the association between AS and hypertension development (Kaess et al., 2012). However, characteristics of the relationship between AS and blood pressure (BP) are still a matter of debate, and this is the focus of two articles in this Research Topic. Obied et al. used a previously validated *in silico*, one-dimensional model of the cardiovascular system to simulate hemodynamic effects and estimate the influence of staged kidney removal on AS measured as PWV. The authors used different configurations to determine PWV measured between different points in the vascular network: Carotid-femoral PWV (cfPWV), carotid-radial PWV (crPWV) and radial-digital PWV (rdPWV)]. Configurations used included the control two kidneys (2KDN), one single kidney (1KDN), no kidneys (0KDN), and a transplanted kidney (TX) attached to the external iliac artery. An increase in PWV and SBP in all configurations *versus* the 2KDN was determined. Since the kidneys are in a high-flow low pressure setting, the absence of functional renal circulation may affect arterial stiffness (AS) and BP, which is certainly observed in clinical studies where reduced renal function is present such as in chronic kidney disease (CKD). Reducing renal mass certainly will affect hemodynamic parameters, and Obied et al. suggest that increases in BP and AS are both a consequence at least at some extent of the necessary blood redistribution taking place during staged reduction in renal blood flow. Obied et al. highlighted the relationships between increased AS and the vascular adaptations in cases of reduced renal blood flow which is relevant given the importance of AS as a risk biomarker for CKD.

## Pulse wave velocity (PWV) and blood pressure in the *db/db* mouse

PWV measures the velocity of pulse transit between two points in a vessel, and it is consequently influenced by several mechanical and functional characteristics of the arteries, including passive mechanical properties, the role of endothelial-derived relaxing factors, and the structure of the extracellular matrix (Townsend et al., 2015). Overall, it has been shown that BP is the most relevant physiological parameter influencing PWV, however the contribution of vessels’ intrinsic and confounding factors to PWV remains to be fully understood. The study by McCallinhart et al. analyzed the relationship between PWV and blood pressure in the *db/db* mouse, a diabetic animal model displaying hyperglycemia, insulin resistance, mild elevations in BP, hypercholesterolemia, and inflammation. Compared to control, these animals showed increased PWV, whereas no differences were observed in aortic intrinsic factors such as diameter, compliance, incremental elastic modulus, stretch ratio, and elastin expression. Interestingly, when the measures of PWV were repeated at equivalent BP levels, the observed differences in PWV disappeared, suggesting that the increased BP might explain the increased PWV and consequently AS. These data are especially pertinent when considering the growing number of studies using PWV in populations with different BP levels or in studies designed to test the effectiveness of antihypertensive drugs (Boutouyrie et al., 2021).

In summary, this Research Topic garnered four compelling research articles related to models in vascular physiology research that highlight their continued ability to disentangle questions related to biomarkers in PVAT, angiogenesis, and vascular stiffness. Without models like the ones included in this Research Topic, answers to challenging research questions would not be possible.
